# ShrimpGPAT: a gene and protein annotation tool for knowledge sharing and gene discovery in shrimp

**DOI:** 10.1186/1471-2164-15-506

**Published:** 2014-06-21

**Authors:** Parpakron Korshkari, Sirintra Vaiwsri, Timothy W Flegel, Sudsanguan Ngamsuriyaroj, Burachai Sonthayanon, Anuphap Prachumwat

**Affiliations:** Center of Excellence for Shrimp Molecular Biology and Biotechnology (CENTEX Shrimp), Faculty of Science, Mahidol University, Rama VI Road, Bangkok, 10400 Thailand; Faculty of Information and Communication Technology, Mahidol University, Salaya Campus, Phutthamonthon District, Nakhon Pathom, 73170 Thailand; National Center for Genetic Engineering and Biotechnology (BIOTEC), National Science and Technology Development Agency (NSTDA), Thailand Science Park, 113 Paholyothin Road, Tambon Khlong 1, Amphoe Khlong Luang, Pathum Thani, 12120 Thailand; Shrimp-Virus Interaction Laboratory, Agricultural Biotechnology Research Unit, National Center for Genetic Engineering and Biotechnology (BIOTEC), National Science and Technology Development Agency (NSTDA), Thailand Science Park, 113 Paholyothin Road, Tambon Khlong 1, Amphoe Khlong Luang, Pathum Thani, 12120 Thailand

**Keywords:** Penaeid shrimp, Decapoda, EST, Transcriptomes, Knowledge base, Community-based functional annotation

## Abstract

**Background:**

Although captured and cultivated marine shrimp constitute highly important seafood in terms of both economic value and production quantity, biologists have little knowledge of the shrimp genome and this partly hinders their ability to improve shrimp aquaculture. To help improve this situation, the Shrimp Gene and Protein Annotation Tool (ShrimpGPAT) was conceived as a community-based annotation platform for the acquisition and updating of full-length complementary DNAs (cDNAs), Expressed Sequence Tags (ESTs), transcript contigs and protein sequences of penaeid shrimp and their decapod relatives and for *in-silico* functional annotation and sequence analysis.

**Description:**

ShrimpGPAT currently holds quality-filtered, molecular sequences of 14 decapod species (~500,000 records for six penaeid shrimp and eight other decapods). The database predominantly comprises transcript sequences derived by both traditional EST Sanger sequencing and more recently by massive-parallel sequencing technologies. The analysis pipeline provides putative functions in terms of sequence homologs, gene ontologies and protein-protein interactions. Data retrieval can be conducted easily either by a keyword text search or by a sequence query via BLAST, and users can save records of interest for later investigation using tools such as multiple sequence alignment and BLAST searches against pre-defined databases. In addition, ShrimpGPAT provides space for community insights by allowing functional annotation with tags and comments on sequences. Community-contributed information will allow for continuous database enrichment, for improvement of functions and for other aspects of sequence analysis.

**Conclusions:**

ShrimpGPAT is a new, free and easily accessed service for the shrimp research community that provides a comprehensive and up-to-date database of quality-filtered decapod gene and protein sequences together with putative functional prediction and sequence analysis tools. An important feature is its community-based functional annotation capability that allows the research community to contribute knowledge and insights about the properties of molecular sequences for better, shared, functional characterization of shrimp genes. Regularly updated and expanded with data on more decapods, ShrimpGPAT is publicly available at http://shrimpgpat.sc.mahidol.ac.th/.

## Background

Marine shrimp in the Family *Penaeidae* have gained status as a very important international seafood trade product of particular economic importance in shrimp farming countries. Despite their economic importance as farmed animals, relatively little is known about the reproduction, immunity and physiology of shrimp when compared to other farmed animals such as poultry and swine. For example, shrimp aquaculture production has been negatively affected by several major pathogens (e.g., white spot syndrome virus and yellow head virus; for reviews, see [1, 2]), and efforts to control these pathogens are impeded by relatively poor knowledge of the shrimp response to them (i.e., shrimp immunity). Although genomic sequences of an organism can yield information about its defense mechanisms, there is currently no completely-sequenced genome for any penaeid shrimp species and only limited characterization of shrimp immune response genes. Similar comments apply to other fields of shrimp biology including reproduction and growth. Shrimp EST collections including recent transcriptomic reads generated by next-generation sequencing (NGS) technologies have helped in shrimp gene and genetic marker discovery (e.g., [3–6]). As such sequencing data are rapidly increasing, and the Shrimp Gene and Protein Annotation Tool (ShrimpGPAT) serves as a platform to extensively collect shrimp molecular sequences for functional annotation and to provide a channel for the shrimp research community to curate and annotate sequences in the form of tags and comments.

Since the first analysis of shrimp ESTs in 1999 [7], several large scale EST studies from various tissues and under various conditions have been carried out for a number of penaeid shrimp species, including the black tiger shrimp *Penaeus (Penaeus) monodon* and the Pacific white shrimp *P. (Litopenaeus) vannamei* (for a review see [8]). Since then, three specialized databases housing shrimp EST sequences have been developed. These are the Marine Genomics Database established in 2005 [9], the *Penaeus monodon* EST Project database established in 2006 [3] and the *Penaeus* Genome database established in 2009 [8]. The Marine Genomics Database includes ESTs and contigs (or “unigenes” as called by the Marine Genomics Database) for four penaeid shrimp species (177,691 EST and 14,726 contig sequences) and also for 23 other marine organisms, such as dinoflagellates, corals, bivalves, crustaceans, sharks, rays, fish, birds, whales and dolphins (314,766 ESTs and 46,421 contigs in total). The Marine Genomics Database plans to include microarray data in a future release. The *Penaeus monodon* EST Project database contains ESTs and contigs (40,001 ESTs and 10,536 contigs) from multiple libraries and tissues of *P. monodon* generated by several laboratories of the Thai shrimp research community. A recent collaboration of shrimp researchers in Thailand and Taiwan resulted in an expansion of *P. monodon* data deposited in the *Penaeus monodon* EST Project database (54,058 ESTs and 12,181 contigs). The *Penaeus* Genome database provides ESTs and contigs for four penaeid shrimp species (196,248 ESTs and 42,332 contigs) and also recently included a genetic linkage map and fosmid library end sequences of *P. monodon*.

Tools available at these three databases include options to search for sequences by BLAST and by homolog descriptions or Gene Ontology terms. All three databases allow users to download sequences of interest. In addition, the Marine Genomics Database currently features both an ability to bookmark sequences for registered users and an EST quality control and submission pipeline for data contributors. The Marine Genomics Database also plans to include a microarray data upload pipeline as well as an automatic incorporation of new ESTs from the Genbank dbEST database in a future version. As EST and contig sequences in these three databases were last updated in 2008–2009, more recently available sequences are not included.

The aim of ShrimpGPAT was to combine multi-source data and include not only EST sequences but also NGS short reads, full-length complementary DNAs (cDNAs) and protein sequences within its data analysis pipeline for sequence quality filtering, contig construction, *in-silico* functional prediction (homolog identification and Gene Ontology prediction) and putative protein-protein interactions. ShrimpGPAT’s tagging and commenting features were designed to allow shrimp research scientists to annotate and provide insights on sequences. ShrimpGPAT initially held a set of ESTs for six decapod species, including four penaeid shrimp. Leekitcharoenphon et al. [10] analyzed and grouped these ESTs into four groups based on homologs found in the genomes of *Drosophila melanogaster* and *Caenorhabditis elegans*, and concluded that this group categorization facilitated functional annotation of shrimp proteomes and their protein sub-populations. Here, we call these categorized groups “reference groups”. Currently, ShrimpGPAT holds full-length cDNA sequences, individual EST sequences, transcript contigs and protein sequences for 14 decapod species (>500,000 combined records) together with putative functional annotations.

## Construction and content

### System design and implementation

**Figure 1:**
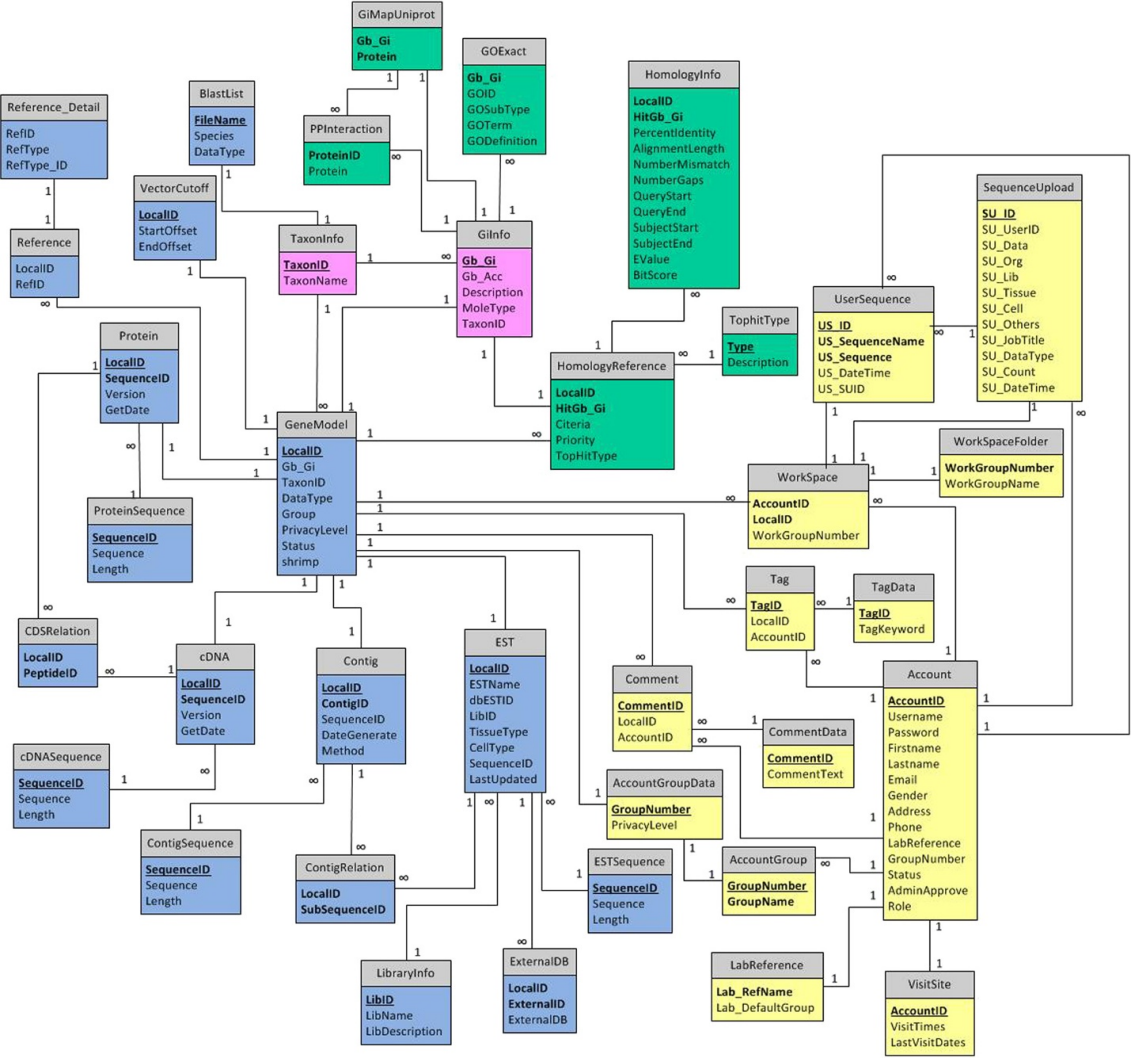
**ShrimpGPAT database schema.** This entity–relationship diagram shows relations among tables of four groups: sequence record tables (blue), *in-silico* annotation tables (green), users’ data tables (yellow) and shared information tables (pink). Primary keys are underlined. Boldface indicates non-null field columns. Connections between tables are represented by lines, and relations between entities are indicated above the connection lines.

ShrimpGPAT was developed as a web-based software environment under Microsoft Windows Server 2008 R2 Enterprise using a relational database of Microsoft SQL Server 2008 SP1 Enterprise for all data storage. Figure 1 shows the ShrimpGPAT relational schema via the entity-relationship diagram, describing the entities and the relationships among all tables as well as the essential keys of all entities of the relations and connections. Tables can be placed roughly into four groups: 1) sequence record tables, 2) *in-silico* annotation tables, 3) users’ data tables and 4) shared information tables (for a detailed description of all tables, see the ShrimpGPAT online documentation). ShrimpGPAT contains a frontend user interface and a backend data analysis pipeline. The user interface was written with the VB.net and ASP.net on HTTP web services with AJAX.net, JQuery and Flash for visualization. The Cytoscape plug-in was used for protein network visualization [11]. Bioinformatic applications currently available to users were integrated with BLAST [12], MUSCLE [13] and MAFFT [14]. The backend data analysis pipeline employed in-house PERL scripts with NCBI E-Utilities [15], NCBI SRA Toolkit [16], phred [17], phd2fasta [18], cross_match [18], BLAST [12], CAP3 [19], Trimmomatic [20] and 454 Sequencing System Software (Newbler and sfffile version 2.8; 454 Life Sciences, Branford, CT) (see below). The processed data (associated information and sequences) were uploaded to the database with ShrimpGPAT data upload tools. The ShrimpGPAT system also supports user authentication and use cases to access the Microsoft SQL database, WorkSpace and community-based functional annotation features.

### Pipeline for in-silico functional annotation

ShrimpGPAT currently focuses on four types of molecular sequences: full-length or partial cDNA, protein, and transcriptomic sequences by both traditional EST cloning and next-generation sequencing technologies. The pipeline for functional annotation comprised four main steps: 1) data acquisition 2) sequence/data cleansing, 3) contig assembly and 4) BLAST plus putative functional annotation. All four steps were applied to EST and NGS short read sequences, but cDNA and protein sequences were not subjected for sequence/data cleansing and contig assembly.

1. Data acquisition

Sequences from GenBank were downloaded by in-house PERL scripts and those from the Marine Genomics database [9] and the *Penaeus monodon* EST Project database [3] were downloaded via their respective websites and by personal communication. The locally-generated EST sequence trace files were processed by phred and phd2fasta into FASTA and .QUAL files. NGS short reads downloaded from the Sequence Read Archive (SRA) were processed by SRA Toolkit. Associated information was formatted for submission to the database by the ShrimpGPAT data upload tools.

2. Sequence/data cleansing

EST sequences were masked by cross_match for vector and contaminating sequences against both full-length vector sequences, if available, and the Univec database [21]. Masked sequences were processed by an in-house PERL script to produce vector-free sequences. Adapter sequences in NGS short reads were trimmed by sfffile or Trimmomatic.

3. Contig assembly

Trimmed sequences were assembled by either CAP3 or Newbler with default parameter settings.

4. BLAST plus putative functional annotation

All nucleotide sequences (EST, transcript contigs and cDNA sequences) were queried (BLASTN and BLASTX) against the nt and nr databases, respectively. BLASTP was performed for protein sequences against the nr database. Homologous sequences were defined as the hits with the following criteria: 1) ≥50% of the query sequence within the aligned region by BLAST, 2) an *E*-value < 10^-6^ (for BLASTN) or < 10^-4^ (for BLASTX and BLASTP), and 3) identity of ≥70% (BLASTN) or of ≥25% (BLASTX and BLASTP).

*Reference sequences and reference groups*: among these homologous sequences of each shrimp sequence query, the overall best homologs (best hits) and the best hits in the *Drosophila melanogaster* or *Caenorhabditis elegans* genomes were selected for each type of BLAST search (BLASTN, BLASTX and BLASTP). Reference sequences were the best hits from BLASTX in *D. melanogaster* if available. If no BLASTX hits in *D. melanogaster* were found, BLASTX hits in *C. elegans* were chosen. If no BLASTX hits were found in either species, overall BLASTX hits were selected. If no BLASTX homologs were found, reference sequences were chosen from BLASTN best hits in a similar manner. For protein sequences, criteria for reference sequences were similar to those for the BLASTX best hits of nucleotide query sequences. Reference groups were assigned by criteria similar to that described in [10].

*Gene Ontology (GO) and protein-protein interactions (PPIs)*: GO classification of each shrimp sequence was derived from its reference proteins described above by mapping with information from the Protein Information Resource [22]. Similarly, putative PPIs were derived through corresponding protein sequences using PPIs from the *Drosophila* Interactions Database [23] and the IntAct molecular interaction database [24].

### Species datasets

Six of the 14 decapod species currently in ShrimpGPAT are penaeid shrimp. The numbers of records along with their scientific and common names are shown in Table 1 (for Record statistics see below). The database will be updated periodically for new sequences and expanded to cover more species.

**Table 1:** **The number of molecular sequence records in ShrimpGPAT**

Species		# of records			
Scientific name	Common name	EST	Transcript contigs ^a^	cDNA	Protein
*Penaeus (Penaeus) monodon*	Black tiger shrimp	86,327	18,410	1,976	602
*Penaeus (Litopenaeus) vannamei*	Pacific whiteleg shrimp	176,592	47,058	74,828	574
*Penaeus (Litopenaeus) setiferus*	White shrimp	1,042	126	135	27
*Penaeus (Fenneropenaeus) chinensis*	Fleshy prawn	10,446	2,714	478	257
*Penaeus (Fenneropenaeus) indicus*	Indian prawn	714	155	348	127
*Penaeus (Marsupenaeus) japonicus*	Kuruma prawn	3,156	662	989	743
*Macrobrachium rosenbergii*	Giant freshwater prawn	4,427	8,550^b^	635	389
*Cherax quadricarinatus*	Cray fish	120	90	239	226
*Pacifastacus leniusculus*	Signal crayfish	802	199	914	88
*Homarus americanus*	American lobster	29,957	12,709	186	227
*Scylla olivacea*	Orange mud crab	203	80	121	0
*Scylla paramamosain*	Green mud crab	3,972	56	720	698
*Callinectes sapidus*	Blue crab	10,563	2,104	173	161
*Carcinus maenas*	Green crab	15,559	7,672	273	275

^a^The number of transcript contigs in each species is the summation of all contig sequences constructed by a set of ESTs and by a set of SRA reads with CAP3 (with default or 97%-similarity parameters) and Newbler (with default parameters).

^b^Including SRA transcript contigs produced by Newbler.

## Utility and discussion

### Data acquisition and sequence analysis pipeline

A curator can obtain a new dataset and formatted records for submission to the *in-silico* functional annotation pipeline. Resulting trimmed ESTs, contig sequences and related putative functions can then be uploaded to the ShrimpGPAT database via ShrimpGPAT data upload tools. Currently, this process is only accessible to designated curators via the administrator mode. Curators must also use this administrator mode to modify an existing record. Registered users can upload and store a limited number of sequences to the ShrimpGPAT database for their private use or to share with the community (see WorkSpace and community-based annotation).

### Record retrieval and sequence analysis tools

**Figure 2:**
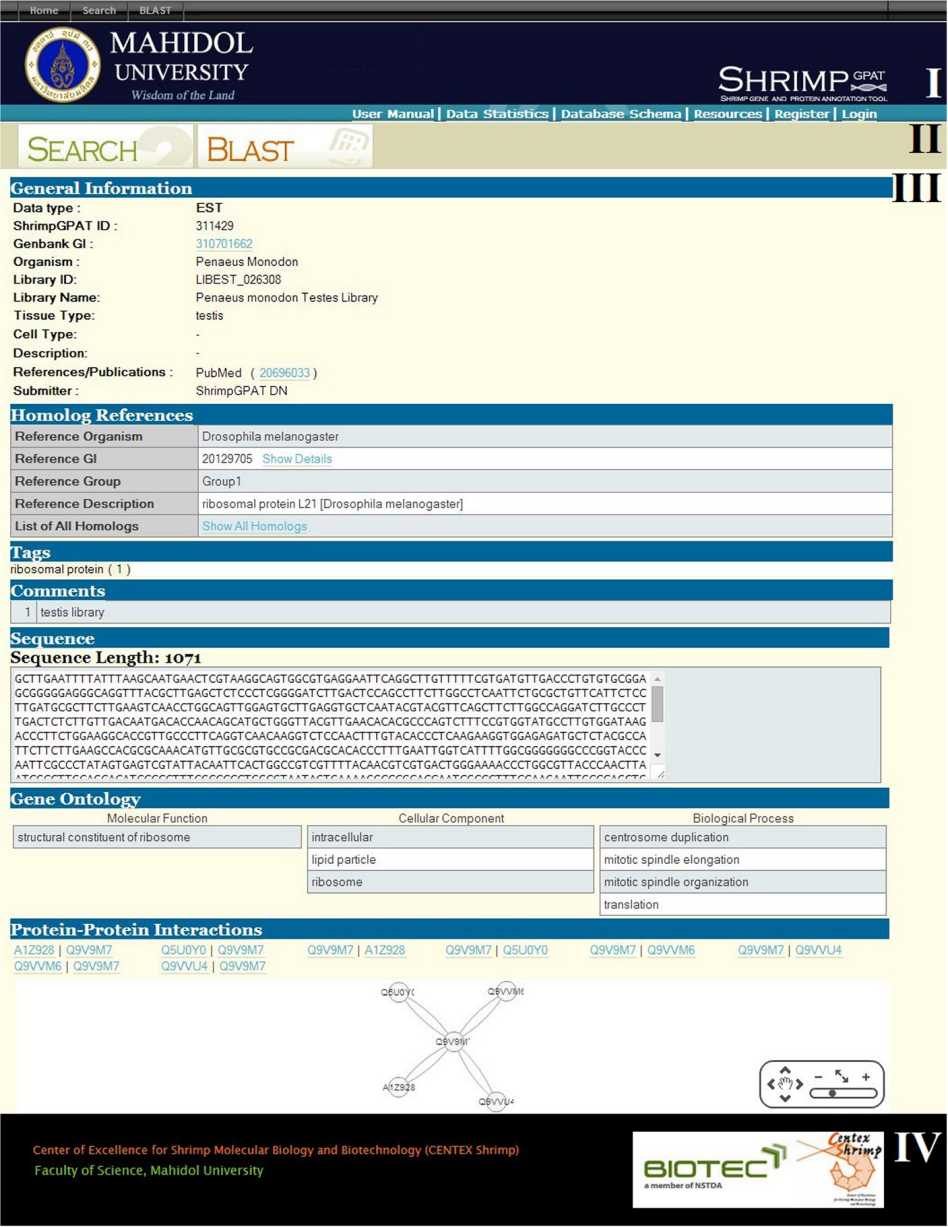
**A screenshot of ShrimpGPAT record display page.** Its layout is divided into I) the title, II) the menu bar, III) the content and IV) the footer. See text for description.

The ShrimpGPAT user interface page contains four areas: title, menu bar, content and footer, arranged from top to bottom as in Figure 2. Title, menu bar and footer areas are relatively static, but the content area displays dynamically-generated information. ShrimpGPAT can be accessed through three main sections listed in the menu bar area, namely Search, BLAST and WorkSpace. The first two can be accessed by any user, but WorkSpace can only be accessed by a registered user (see below). Records can be retrieved either by a keyword text search (Search button) or by a sequence query (BLAST button). Two types of keyword text search are currently permitted: free text search and advanced search for specified fields. The BLAST search function is set with default parameters but with options for several *E*-value cutoffs. Records returned by both Search and BLAST are displayed in the same format for easy viewing and investigation. Users can select records for further analysis through searching with BLAST, creating Multiple Sequence Alignments (MSA), exporting sequences in a FASTA file, bookmarking to their private WorkSpace or adding of tags or comments. ShrimpGPAT currently provides two sets of sequence analysis tools in sections where such analyses are applicable: BLAST and MSA. BLAST is parameterized to a default setting, except for *E*-value cutoffs, and MSA provides MAFFT and MUSCLE analyses with default parameter settings.Records in a result list from any executed queries can be investigated further by clicking on a ShrimpGPAT ID, which will display full information regarding that particular record, e.g., sequence type, organism, tissue, organ of expression, references/publications as well as external database IDs (Figure 2). External database IDs are hyperlinked to corresponding external database records. Homolog information (reference sequences and reference groups) is displayed below the general information. Note that only one reference sequence is displayed on this page, but clicking on the hyperlinks “Show Details” or “Show All Homologs” reveals all reference sequences or homologous sequences with a complete BLAST result. Tags, comments, sequence characters of a record, GO and putative PPIs are consecutively displayed below the homolog information section.

### WorkSpace and community-based annotation

WorkSpace and community-based annotation features are reserved for registered users. ShrimpGPAT WorkSpace provides private space for records of interest. Within WorkSpace, a user can create virtual folders to store records and can later delete or rename the folders. Records can be moved between or copied into virtual folders. Records stored in WorkSpace can be used later for additional sequence analyses or for sequence downloading. Importantly, users can help annotate records with tags and comments (ShrimpGPAT community-based annotation). Tags are short keywords, but comments can be long strings of text. These tags and comments are publicly displayed for text search to any users, so they enable knowledge sharing among the shrimp research community. For example, users can input gene names as tags and information of references/publications as comments. However, some well-known shrimp gene names known by abbreviations such as PmRab7, may not be present as such in description lines of GenBank full-length cDNA or protein records but instead be written in full, i.e., “Penaeus monodon Rab7”. Thus, a search using “PmRab7” might fail, while a search using “Penaeus monodon Rab7” or just “Rab7” would succeed. Thus, users can easily retrieve records with gene names if such records are tagged with corresponding gene names, but if no records are retrieved, name variations can be tried. Usage of tags and comments may be added to expand tags for a particular sequence or add them to sequences that are currently uncharacterized in the database but may later be studied and given gene names. Users can also share their dataset with the community via the ShrimpGPAT data upload tool to deposit the data as permanent records. Similarly, users can upload sequences for their private use, but such private sequences will be stored in user’s virtual folders for a period of only three months.

### Record statistics

Table 1 shows the number of molecular sequence records for the 14 decapods currently available in the ShrimpGPAT database. *P. vannamei* has the highest number of records (~299,000), and *P. monodon* has the second highest (~138,000). The numbers signify their importance as species of the highest interest to the shrimp scientific research community and species most-cultivated or captured for trade. Similarly, the six penaeid shrimp have combined records that number about four times that of the other eight decapod species in the database (i.e., ~460,000 *vs.* 111,000). A large proportion of the records for each species are ESTs and transcript contigs, whereas the numbers of cDNA and protein records are still relatively small. The number of transcript contigs for each species is the summation of all contig sequences constructed by the set of ESTs and by the set of SRA reads. Note that transcript contig records produced by different contig assemblers (e.g., CAP3 and Newbler) may constitute the same sequences. Regarding transcript contigs of SRA reads, *Macrobrachium rosenbergii* is the only species that currently has transcript contigs derived from an SRA dataset (81,411 reads for 50 million base pairs; [6]). Soon, SRA transcript contigs for other species will be available, e.g., *P. vannamei* with eight NGS runs in the SRA database, constituting 80 million reads for 7.9 billion base pairs. Among the 14 species, *Scylla olivacea* has the lowest number of records in its EST collection. It is the first publicly-available collection of ESTs for this species and it was recently generated by our laboratory. The current release of the database contains full-length cDNA and protein sequences downloaded from GenBank as of July 2013. Thus, sequences of some known shrimp genes might not currently be in the ShrimpGPAT database because 1) they were not present in GenBank at the time of the most recent download, 2) they were reported only in papers without a submission to GenBank, or 3) they were deposited elsewhere. Such sequences can be manually added by designated curators or gradually submitted and reported by users. Complete descriptive statistics and sources of ShrimpGPAT records are available on the ShrimpGPAT statistics page.

### New and improved features for the shrimp community

ShrimpGPAT provides new and improved features that are lacking in the three existing specialized genomic databases for shrimp. First, ShrimpGPAT provides sequences of full-length cDNAs, proteins and transcript contigs from the rapidly growing number of NGS reads, in addition to traditional EST sequences that are provided by the existing databases. Its *in-silico* functional annotation pipeline can readily facilitate new data. Currently, ShrimpGPAT holds the highest number of molecular sequence records and species of penaeid shrimp (6 *vs.* 4 species in the Marine Genomics Database) and their decapod relatives (8 *vs*. 4 species in the Marine Genomics Database). Second, in terms of *in-silico* functional annotation features, putative sets of protein-protein interactions and reference sequences (reference groups) can only be found in ShrimpGPAT. Reference sequences are homologs in the genomes of *D. melanogaster* and *C. elegans* (decapods’ closest relatives whose genomes are better characterized). Most existing databases provide only best-hit homologous sequences (which may or may not be those in the genomes of *D. melanogaster* and *C. elegans*), while ShrimpGPAT provides all homologous sequences that meet our criteria (see above). Similar to the other databases, GO classification is provided. Third, the unique set of tools available in ShrimpGPAT includes multiple sequence alignment, WorkSpace and community-based annotation. WorkSpace allows users to keep records of interest and their uploaded sequences. Users can upload sequences to share with others or use privately. Users of ShrimpGPAT can also utilize a set of tools similar to those found in the three existing databases (i.e., text search, BLAST and sequence download). With a large and expanding data set and its new features, ShrimpGPAT provides a more comprehensive database with more easily accessible tools than those of the three existing databases mentioned above. To the best of our knowledge ShrimpGPAT is only shrimp database that offers community-based annotation with tags and comments.

## Conclusions

ShrimpGPAT is a new online resource to help shrimp researchers investigate molecular sequences of penaeid shrimp and their decapod relatives. ShrimpGPAT provides shrimp biologists with easy access to a comprehensive collection of rapidly growing sequence information. The database will be periodically updated and expanded to cover more crustacean species with its *in-silico* functional annotation pipeline. It is envisioned that collaborative knowledge built via community-based annotation will rapidly accelerate shrimp gene discovery and research.

## Availability and requirements

ShrimpGPAT is publicly available via the Website URL http://shrimpgpat.sc.mahidol.ac.th/. Registration requires a valid email address. The initial dataset based on Leekitcharoenphon et al. [10] can be accessed at http://shrimpgpat.sc.mahidol.ac.th/v1/.

## Abbreviations

AJAX: Asynchronous JavaScript and XML
BLAST: Basic local alignment search tool
cDNA: Complementary DNA
EST: Expressed sequence tag
GO: Gene Ontology
MSA: Multiple sequence alignments
NGS: Next-generation sequencing technology
PPI: Protein-protein interaction.
